# The *Sporomusa* type Nfn is a novel type of electron-bifurcating transhydrogenase that links the redox pools in acetogenic bacteria

**DOI:** 10.1038/s41598-020-71038-2

**Published:** 2020-09-10

**Authors:** Florian Kremp, Jennifer Roth, Volker Müller

**Affiliations:** Department of Molecular Microbiology and Bioenergetics, Institute of Molecular Biosciences, Johann Wolfgang Goethe University, Max-von-Laue Str. 9, 60438 Frankfurt, Germany

**Keywords:** Oxidoreductases, Bacterial physiology, Metabolism

## Abstract

Flavin-based electron bifurcation is a long hidden mechanism of energetic coupling present mainly in anaerobic bacteria and archaea that suffer from energy limitations in their environment. Electron bifurcation saves precious cellular ATP and enables lithotrophic life of acetate-forming (acetogenic) bacteria that grow on H_2_ + CO_2_ by the only pathway that combines CO_2_ fixation with ATP synthesis, the Wood–Ljungdahl pathway. The energy barrier for the endergonic reduction of NADP^+^, an electron carrier in the Wood–Ljungdahl pathway, with NADH as reductant is overcome by an electron-bifurcating, ferredoxin-dependent transhydrogenase (Nfn) but many acetogens lack *nfn* genes. We have purified a ferredoxin-dependent NADH:NADP^+^ oxidoreductase from *Sporomusa ovata*, characterized the enzyme biochemically and identified the encoding genes. These studies led to the identification of a novel, *Sporomusa* type Nfn (Stn), built from existing modules of enzymes such as the soluble [Fe–Fe] hydrogenase, that is widespread in acetogens and other anaerobic bacteria.

## Introduction

The Wood–Ljungdahl pathway (WLP) is an ancient pathway that combines carbon dioxide fixation with the synthesis of ATP and, therefore, is considered as one of the oldest life sustaining pathways on Earth^1,2^. CO_2_ is reduced in two branches (the carbonyl- and the methyl-branch) to CO and a corrinoid-bound methyl group, respectively, by several consecutive enzymatic steps. Subsequently, CO and the methyl group are condensed and further converted to acetate via acetyl phosphate^3,4^. Acetogenic bacteria are phylogenetically very diverse but the basic chemistry of the WLP is identical. In every acetogen studied so far, ATP is synthesized by substrate level phosphorylation in the acetate kinase reaction, but additional ATP synthesis by a chemiosmotic mechanism is absolutely required for a net ATP synthesis^5,6^. Acetogens differ in the respiratory enzymes used: some use a ferredoxin:NAD^+^ oxidoreductase (Rnf)^7–9^, other a ferredoxin:H^+^ oxidoreductase (Ech) as respiratory enzyme^10^. The ion translocated by these enzymes can be either a proton or a sodium ion^11–13^.

During lithotrophic growth electrons for CO_2_ reduction are ultimately derived from molecular hydrogen. Therefore, acetogens employ hydrogenases and, again, there is great variety of hydrogenases used: soluble, electron-bifurcating [Fe–Fe] hydrogenases^14^ and membrane-bound [Ni–Fe] hydrogenases^10^. Along with the diversity of hydrogenases goes the diversity of electron carriers involved in hydrogen oxidation which can be NAD^+^, NADP^+^ and/or ferredoxin^15–18^. The same is true for the reduction steps in the WLP: in some organisms, ferredoxin (Fd) and NAD^+^ are the major electron carriers, in others NADP^+^ is also involved^5^. Since electron carriers coupled to oxidation reactions may be different from electron carriers involved in the reductive branch, a redox balancing module is essential to produce the correct electron carriers in the right amount for the WLP^6^. Reduction of ferredoxin is highly endergonic with electrons coming from hydrogen and the energy barrier is overcome either by a soluble energy-coupled, electron-bifurcating hydrogenase that uses NAD^+^ as cooxidant^18^ or by a membrane-bound enzyme, the Rnf complex, that uses reverse electron transfer to drive endergonic ferredoxin reduction with NADH as reductant^9^. NADP^+^ reduction with NADH is also endergonic but acetogens studied so far lack a membrane-bound transhydrogenase; they use electron bifurcation to overcome the energetic barrier by an NADH-dependent reduced ferredoxin:NADP^+^ oxidoreductase (known as Nfn in acetogens)^19^.

Interestingly, some acetogens lack *nfn* genes and must have a different enzyme to reduce NADP^+^. To find this missing link, we have studied the metabolism of the acetogen *Sporomusa ovata* and will provide evidence for a novel type of NADH-dependent reduced ferredoxin:NADP^+^ oxidoreductase built from existing redox modules.

## Results

### Insights into the central metabolism of *S. ovata*

The genome of *S. ovata* was sequenced in 2013 by Poehlein et al.^20^ (accession no.: ASXP00000000). Almost all proteins of the methyl- and the carbonyl branch were found to be encoded in one gene cluster (SOV_1c07560- SOV_1c07730, Supplementary Fig. 1), but the genes encoding a SeCys-containing formate dehydrogenase (Fdh), phosphotransacetylase (Pta) and acetate kinase (Ack) are located somewhere else in the genome (SOV_1c07830 + SOV_1c07840, SOV_1c07460 and SOV_1c10930). A search for redox balancing modules revealed genes encoding an electron-bifurcating [Fe–Fe] hydrogenase (SOV_1c07930-SOV_1c07970) and an Rnf complex (SOV_1c08080-SOV_1c08130) but genes encoding an Nfn-type transhydrogenase were not found. Therefore, the basic metabolism of *S. ovata* is similar to that of Rnf complex-containing acetogens like the model acetogen *A. woodii*^5^. To get a more precise model of the metabolism, the redox carriers involved in the WLP were determined experimentally. To this end, *S. ovata* was grown and harvested in late exponential growth phase and cells were disrupted by a French pressure cell. After cell debris was removed, membranes were separated from the cytoplasmic fraction by ultracentrifugation and enzyme activities were measured as described in methods. The membrane fraction catalysed ferredoxin-dependent NAD^+^ reduction (0.14 U/mg), NADP^+^ was not reduced (Supplementary Fig. 2a, 2b and 2c). Unlike *A. woodii* but like *M. thermoacetica*, the methylene-THF dehydrogenase (MTHFDH), which was measured in the cytoplasmic fraction, did not reduce NAD^+^ but NADP^+^ (33 U/mg) (Supplementary Fig. 3a and 3b). These findings show that *S. ovata* uses both, NADH and NADPH as reductants in the WLP but since there is neither an Nfn complex nor a membrane-bound transhydrogenase encoded in the genome of *S. ovata*, the question arose of how the NADH- and Fd-pools are coupled to the NADPH-pool. Transhydrogenases are described to have a high diaphorase activity and, therefore, we decided to search for and enrich an NADPH:methyl viologen (MV) oxidoreductase activity from the cytoplasmic fraction.

### Purification and analysis of an NADPH:MV oxidoreductase

**Figure 1:**
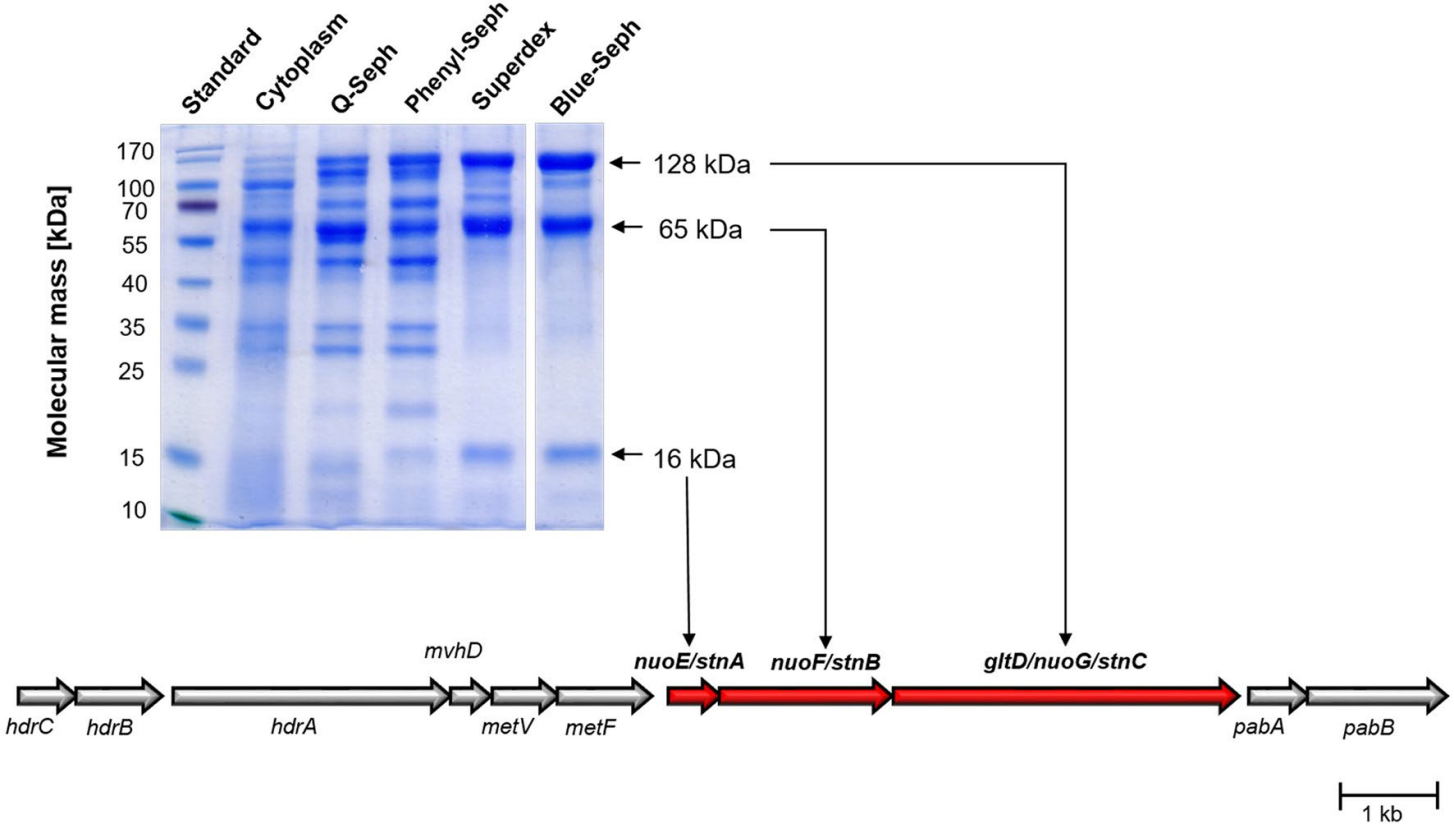
Purification of the *Sporomusa* type Nfn complex of *S. ovata*. 10 µg of protein sample from each purification step were separated in a denaturating SDS-PAGE. MALDI-TOF-MS analysis of the proteins with the apparent molecular masses of 128, 65 and 16 kDa identified the encoding genes to be located downstream of the MTHFR encoding genes (*metVF*) and upstream of *pabAB* which encode an aminodeoxychorismate/anthranilate synthase. The Stn encoding genes were annotated to code for Nuo-like proteins (*nuoE, nuoF* and *nuoG).* Further, *stnC* was annotated to code for a protein which is in part similar to GltD, the small subunit of glutamate synthases.

The cytoplasmic fraction of *S. ovata* catalysed NADPH:MV oxidoreductase with an activity of 3 U/mg. The activity was enriched 93-fold by fast liquid chromatography (FPLC) on Q-Sepharose, Phenyl-Sepharose, Superdex 200 and Blue-Sepharose from 3 to 278 U/mg (Supplementary Table 1). Three major proteins with molecular masses of 128, 65 and 16 kDa became visible after separation of the preparation by SDS-PAGE (Fig. 1). To analyse whether these proteins are part of a single protein complex, the enriched sample was separated by native PAGE which revealed three protein complexes with molecular masses of approximately 232, 142 and 91 kDa (Supplementary Fig. 4a). The protein complexes were cut out of the gel, treated with 2% SDS and 0.67% mercaptoethanol in 60 mM Na_2_CO_3_ and separated in a denaturating SDS-PAGE (Supplementary Fig. 4b). The protein complex with a molecular mass of 232 was separated into the three proteins seen before (128, 65 and 16 kDa), showing that they are part of a single complex. The protein complex with a molecular mass of 142 kDa contained two major proteins of 128 and 65 kDa, indicating decomposition of the complex during native PAGE. The third protein complex with a size of 91 kDa contained two proteins with apparent molecular masses of 65 and 72 kDa, indicating a minor contamination in the preparation. This is corroborated by the observation that a 72 kDa protein is not consistently observed in the preparations. Since the complex was shown to decompose during gel electrophoresis the molecular mass was determined by analytical gel filtration on Superdex 200; it amounted to approximately 840 kDa, consistent with the mass of a tetrameric heterotrimer. The proteins with a molecular mass of 128, 65 and 16 kDa were identified by MALDI-TOF-MS analysis to be encoded by the three consecutive genes *SOV_1c07740-SOV_1c07760* which are located downstream of the WLP encoding gene cluster (Fig. 1). SOV_1c07740-SOV_1c07760 are annotated as NuoE-, NuoF- and GltD/NuoG-like proteins.

### Activities and basic properties of the reactions catalysed by the purified enzyme

The purified protein complex exhibited not only NADPH:methyl viologen but also NADPH:benzyl viologen (BV) activity (238.0 U/mg). Besides, NADH can be used as electron donor for MV and BV reduction (0.33 U/mg and 0.23 U/mg), indicating a possible function as transhydrogenase which connects the NADPH with the NADH-pool. Indeed NAD^+^ could be reduced with electrons from NADPH, albeit with low activity (0.06 U/mg). Furthermore, FAD (3.2 U/mg) and FMN (5.2 U/mg) were reduced with NADPH as reductant, demonstrating that flavin molecules can bind to the Stn complex, which is a key feature of electron-bifurcating enzymes. The complex reduced ferricyanide (315 U/mg) with NADPH, indicating that the Stn complex also uses iron containing electron donors/acceptors, besides pyridine nucleotides. Next, we tested if Fd (reduced by CODH), which is used by electron-bifurcating enzymes as low potential electron acceptor, can serve as electron donor for the energetic “downhill” electron transport to NADP^+^ or NAD^+^ and, indeed, NADP^+^- and NAD^+^ reduction was observed with 0.65 and 0.23 U/mg, respectively. Since Fd, NADP(H) and NAD(H) are used as electron donors/acceptors, we tested if the Stn complex uses the mechanism of flavin-based electron bifurcation (FBEB). Therefore, simultaneous reduction of NAD^+^ and Fd with NADPH was tested and, indeed, reduction of NAD^+^ was found with a maximum activity of 4.5 U/mg and Fd was reduced with a maximal activity of 3.0 U/mg under optimal conditions of 50 °C and pH 8 (Supplementary Fig. 5a and 5b). Reduction of NAD^+^ did not stop after ferredoxin was fully reduced, as it was observed for the electron-bifurcating hydrogenase of *Desulfovibrio fructosovorans*, indicating reoxidation of Fd_red_^21^. K_m_-values were determined to be 39 µM for NADPH and 59 µM for NAD^+^ (Supplementary Fig. 6a and 6b). Further, the enzyme catalysed Fd_red_- and NADH-dependent reduction of NADP^+^ with a maximal activity of 5.0 U/mg and half maximal activity was found with concentration of 31 µM NADP^+^ or 40 µM NADH (Supplementary Fig. 6c and 6d). Addition of FMN (50 µM) to the bifurcation assay stimulated the NAD^+^- and Fd reduction ~ 3–4-fold to 17 U/mg and 10 U/mg, respectively. NADP^+^ reduction with Fd_red_ and NADH was stimulated ~ eightfold to 42 U/mg. Also FAD (50 µM) stimulated the activities but to a smaller extent.

**Figure 2:**
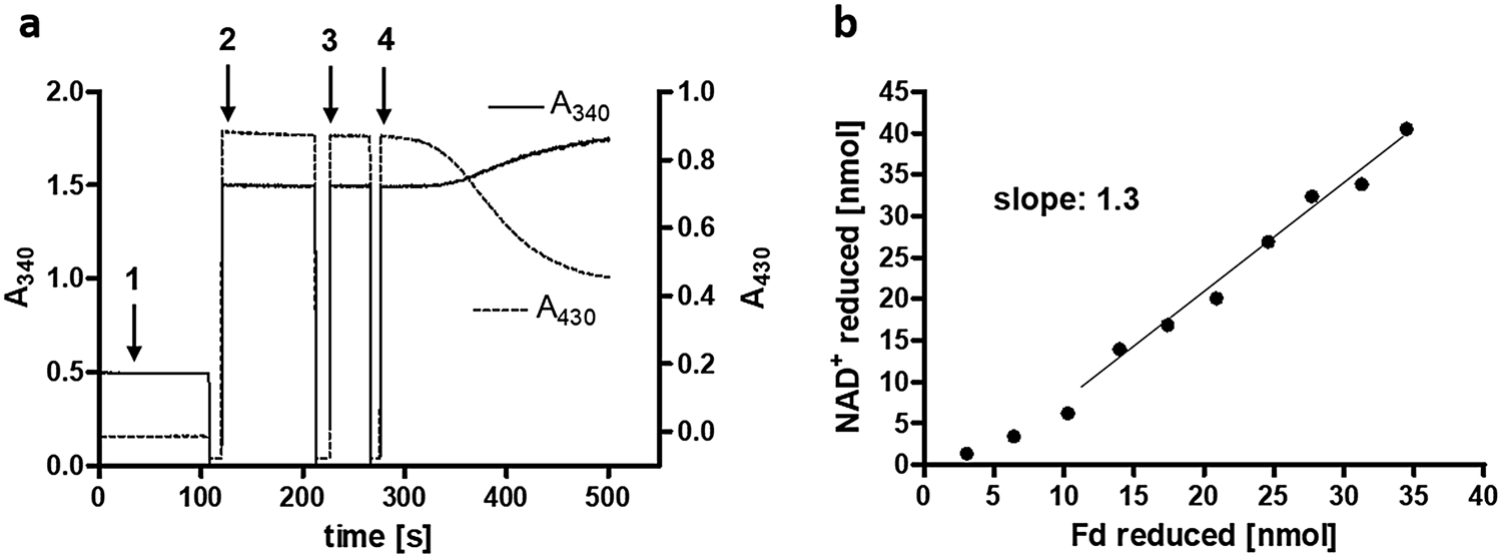
Stoichiometry of NAD^+^ and Fd reduced. (**a**) 0.4 mM NADP^+^ was reduced with glucose-6-phosphate dehydrogenase and 40 mM glucose-6-phosphate to ensure a constant level of NADPH during the assay (1). 180 µM Fd and 1 mM NAD^+^ were added (2 and 3) and the assay was started by addition of 6.5 µg protein (4). The reduction of NAD^+^ and ferredoxin were followed at 340 nm and 430 nm, respectively. The amount of NAD^+^ and ferredoxin reduced was calculated from (**a**) in 5-s intervals and applied in (**b**) to calculate the ratio of NAD^+^ reduced to ferredoxin reduced.

**Figure 3:**
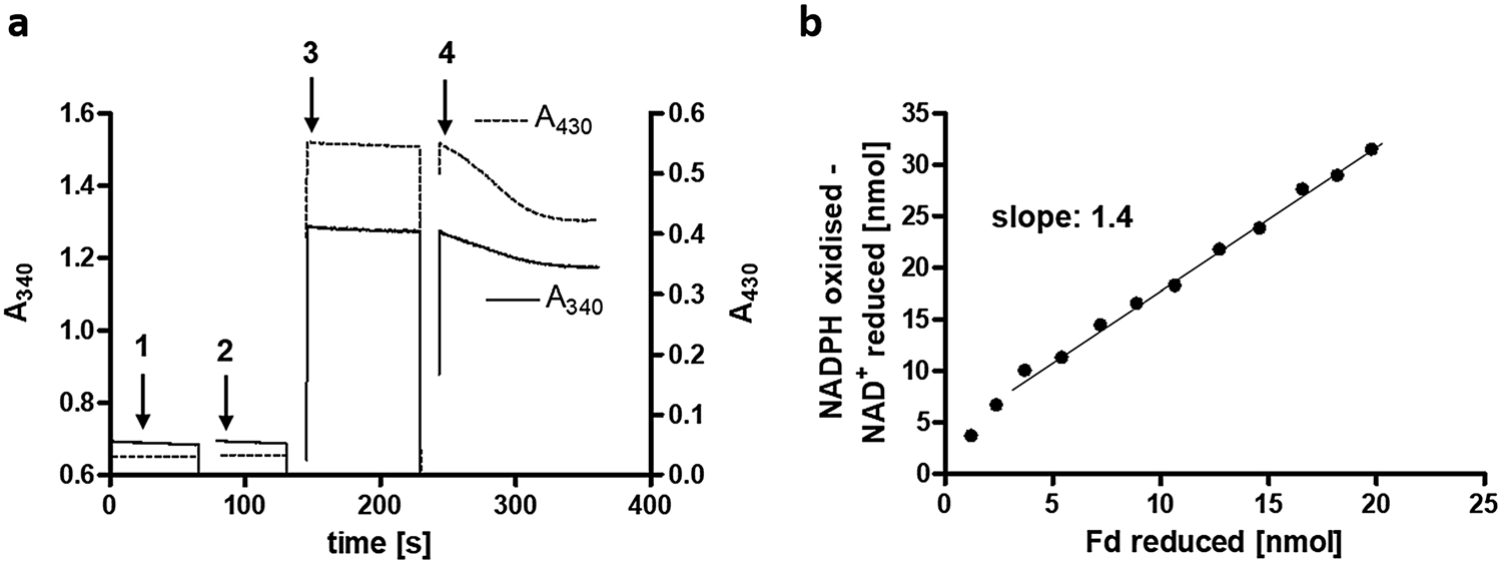
Stoichiometry of NADPH oxidised per Fd reduced. (**a**) 0.5 mM NADPH (1), 1 mM NAD^+^ (2) and 120 µM Fd (3) were added to the buffer and the assay was started with 9.75 µg protein (4). The oxidation of NADPH and the reduction of ferredoxin were followed at 340 nm and 430 nm, respectively. The amount of NADPH oxidised and ferredoxin reduced were calculated from (**a**) in 5-s intervals and applied in (**b**) to calculate the ratio of NADPH oxidised minus NAD^+^ reduced to ferredoxin reduced.

The experiments described above are consistent with the hypothesis that the complex catalyses NADH-dependent NADP^+^ reduction with simultaneous oxidation of reduced ferredoxin as energetic driving force by flavin-based electron confurcation. To determine the stoichiometries of the reactants involved, the reverse reaction was analysed. In order to run the reaction for a longer period the standard assay was altered: The level of NADPH was kept constant with an NADPH regeneration system (0.4 mM NADP^+^, 40 mM glucose-6-phosphate and 1 U glucose-6-phosphate dehydrogenase) and 180 µM Fd and 1 mM NAD^+^ were used in the assay. The stoichiometry of NAD^+^ reduced per ferredoxin reduced was calculated to be 1.3 from the concentrations of NADH and Fd_red_ measured in 5-s intervals (Fig. 2a, b). Under completely coupled conditions NAD^+^ and Fd are expected to be reduced in a 1:1 stoichiometry according to Eq. 1:

1 $${\text{2 NADPH }} + {\text{ NAD}}^{ + } + {\text{ Fd}}_{{{\text{ox}}}}\rightarrow {\text{2 NADP}}^{ + } + {\text{ NADH }} + {\text{ Fd}}_{{{\text{red}}}}$$

but since the complex also catalyses NAD^+^ reduction with only NADPH or Fd_red_ as electron donor and reoxidation of Fd_red_ occurs, the deviation can be explained. Ratios of NADPH oxidised to Fd reduced were calculated to be 1.4 by using the same assay without NADPH regeneration system, but 0.5 mM NADPH instead and only 120 µM Fd (Fig. 3a, b). NADH and NADPH both absorb at 340 nm and have the same extinction coefficient, therefore the expected ratio of NADPH oxidised per Fd reduced of 2 (Eq. 1) was adjusted, by the theoretical amount of NAD^+^ reduced, to 1. Again, deviation from the theoretical value is expected, since the complex also couples the individual reactions.

In sum, the purified NADH:MV oxidoreductase from *S. ovata* is a novel type of Nfn complex that catalyses NADH-dependent Fd_red_:NADP^+^ oxidoreductase activity. Therefore, we propose the name *Sporomusa* type Nfn (Stn) for the heterotrimeric protein complex, comprising of the 16 kDa subunit StnA, the 65 kDa subunit StnB and the 128 kDa subunit StnC. Because StnC was also annotated as formate dehydrogenase, formate oxidation with methyl viologen as electron acceptor was tested, but no activity was observed.

### In silico analysis of the genes coding for Stn and basic biochemical properties

**Figure 4:**
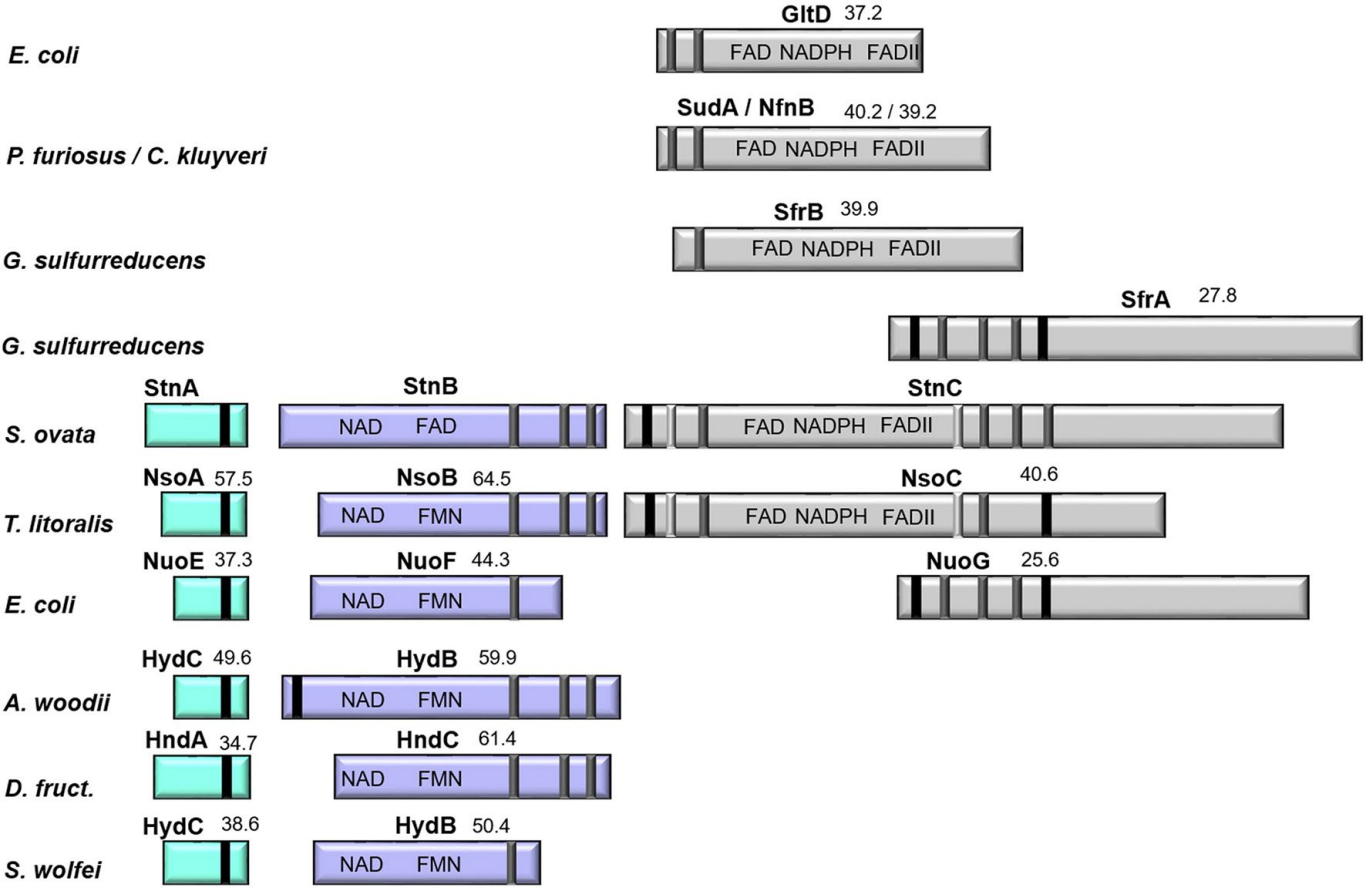
Comparison of Stn subunits to homologous proteins. StnA is a homolog of NuoE-like proteins and StnB belongs to the NuoF-like protein family both known to be part of electron-bifurcating enzymes such as the bifurcating hydrogenases of several organisms (e.g. *A. woodii*). StnC seems to be a fusion of a GltD-like and a NuoG-like protein. The family of GltD-like proteins has also members, participating in electron bifurcation (e.g. NfnB of *C. kluyveri*). The depicted proteins are glutamate synthase small subunit GltD of *E. coli*, the homologous subunits SudA and NfnB from sulfide dehydrogenase and NADH-dependent reduced ferredoxin:NADP^+^ oxidoreductase of *P. furiosus* and *C. kluyveri*^19,29,30^, the subunits SfrB and SfrA from the soluble Fe(III) reductase of *G. sulfurreducens*^32^*,* the subunits NsoA, NsoB and NsoC of the NADPH-dependent sulfur oxidoreductase from *T. litoralis*^22^*,* NuoE, NuoF and NuoG from the NADH-binding module of complex I from *E. coli*^24^, the subunits HydC and HydB (HndA and HndC) from the electron-bifurcating hydrogenase of *A. woodii* and *D. fructosovorans*^18,21^, respectively, and the subunits HydC and HydB from the non-bifurcating hydrogenase of *S. wolfei*^23^*.* Numbers indicate sequence identity on protein level. Black bars represent [2Fe–2S]-clusters, grey bars represent [4Fe–4S]-clusters and white bars represent cysteine-rich sequences known from Nso^22^. FAD, FMN and NAD(P)-binding sites are indicated.

Sequence comparison using BlastP algorithm revealed 57.5, 49.6, 38.6, 37.3 and 34.7% identity of StnA to subunit NsoA of the NADPH-dependent sulfur/oxygen oxidoreductase from *Thermococcus litoralis*^22^, HydC of the electron-bifurcating hydrogenase from *A. woodii*^18^ and the NAD(H)-dependent hydrogenase of *Syntrophomonas wolfei*^23^, NuoE of complex I from *E. coli*^24^ and HndA of the electron-bifurcating hydrogenase from *D. fructosovorans*^21,25^, respectively (Fig. 4). StnA is predicted to have a molecular mass of 19 kDa and a thioredoxin-like domain with conserved [2Fe–2S]-binding motif (Supplementary Fig. 7). StnB is a homolog of the subunits NsoB, HndC, HydB and NuoF from the same protein complexes, sharing 64.5, 61.4, 59.9, 50.4 and 44.3% identity, respectively. StnB has a predicted molecular mass of 63.5 kDa and the GxGxxG motif indicative for NADH-binding^26,27^ as well as the FMN-binding region^28^ are conserved. Additionally, the [4Fe–4S]-cluster binding residues of NuoF are conserved in StnB (Supplementary Fig. 8). As in StnA the N-terminus is proposed to form a thioredoxin-like domain, but unlike in StnA 2 of the 4 cysteines responsible for [2Fe–2S]-cluster binding are changed to glycine. The C-terminus of StnB has two more predicted [4Fe–4S]-binding sites that are also found in HydB and NsoB. StnC seems to be a fusion of an N-terminal GltD-like domain and a C-terminal NuoG-like domain. NsoC also combines these two domains and shows 40.6% identity to StnC. Further the N-terminal domain of StnC shows identities of 40.2, 39.9, 39.2 and 37.2% to subunit SudA from a sulfide dehydrogenase/ferredoxin:NADPH oxidoreductase of *Pyrococcus furiosus*^29–31^, subunit SfrB of the NADPH:Fe^3+^ oxidoreductase from *Geobacter sulfurreducens*^32^, the NADPH-binding subunit NfnB from the Nfn complex of *C. kluyveri*^19^ and subunit GltD of the glutamate synthase of *E. coli*, respectively. The C-terminal domain of StnC is slightly similar to subunit SfrA from *G. sulfurreducens* and NuoG from *E. coli* showing identities of 27.8 and 25.6%. StnC is predicted to have a molecular mass of 126.4 kDa and a proposed N-terminal [2Fe–2S]-ferredoxin domain, but only 5 of 8 FeS coordinating residues from SudA, SfrB and GltD are conserved in StnC (Supplementary Fig. 9). Two glycine-rich regions are found in StnC. The first is proposed to be part of an FAD-binding motif^27^, whereas the second motif GxGxxA is proposed to bind NADP(H) rather than NAD(H)^33^. The C-terminal domain has a proposed [4Fe-4S]-dicluster domain and a [4Fe–4S]-cluster of the molybdopterin oxidoreductase family (Supplementary Fig. 10). Furthermore, two cysteine-rich motifs (HxxxCxxxCxxxCP and CxxCxCxxxxC), which were proposed to be a product of the integration of GltD into a NuoG-like protein^22^, are conserved in StnC but whether they bind FeS-cluster is not known so far. In sum, the Stn complex is predicted to bind 32 mol of Fe and sulfur and one mol FMN and FAD per heterotrimer. Using the method of Fish^34^ 35.5 ± 2 mol of Fe were determined per mol of protein (n = 8), indicating that at least one of the two before mentioned cysteine-rich motifs in StnC might bind an FeS-cluster. Contrary to our expectations, after precipitation of the protein and subsequent separation of the flavin-containing supernatant by thin layer chromatography (TLC), FAD was identified to be the only flavin bound (Supplementary Fig. 11). The exact amount of bound FAD could not be determined since flavins are only loosely bound and need to be added to the buffers during purification to prevent loss of protein activity.

### Biochemical properties of heterologously produced StnC

The genes *stnA*, *stnB* and *stnC* encoding the single subunits were cloned between the NheI and BamHI restriction sites of pET21a adding a strep-tag encoding sequence at the N-terminus. The plasmids pET21a_strepStnA, pET21a_strepStnB and pET21a_strepStnC were transformed in *E. coli* BL21 (DE3) Δ*iscR* and produced according to Demmer et al.^35^. Production of strep-tagged StnA and StnB resulted in the formation of inclusion bodies, hence further biochemical analysis was not possible. Strep-tagged StnC was purified to apparent homogeneity using one step purification on Strep-Tactin and an additional purification step using FPLC on Superdex 200. The purified protein exhibited NADPH oxidation with methyl viologen, FAD, FMN and ferricyanide with activities of 58, 0.7 and 4.5, 16 U/mg, respectively. The identity of bound Flavin was elucidated by TLC to be FAD.

### Model of acetogenesis from H_2_ + CO_2_ in ***S. ovata***

**Figure 5:**
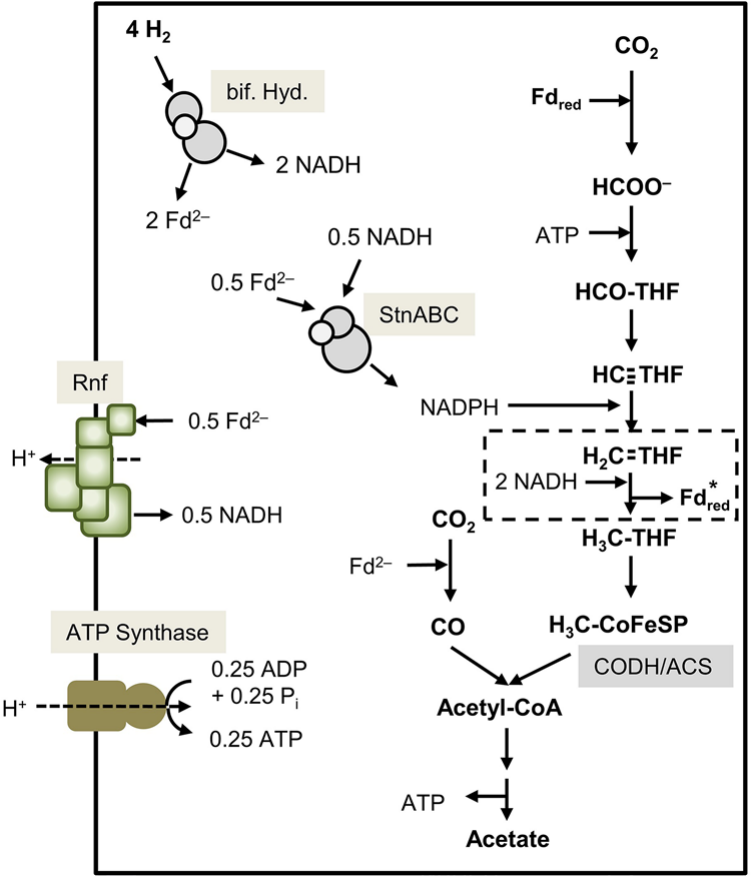
Biochemistry and bioenergetics of acetogenesis from H_2_ + CO_2_ in *S. ovata.* For explanations, see text. *The MTHFR is proposed to be electron-bifurcating. The second electron acceptor is assumed to have similar redox properties to that of Fd. For simplicity reasons we suppose Fd as second electron acceptor in our model. Abbreviations: bif. Hyd., electron-bifurcating hydrogenase; Stn, *Sporomusa* type Nfn.

The results of this study lead to a genome-based model of the central metabolism of *S. ovata*, which is expanded by biochemical analysis. The formate dehydrogenase shows highest similarity to the Fd-dependent Fdh of *C. pasteurianum* and therefore Fd is assumed as electron donor for CO_2_ reduction^36^. The MTHFDH was experimentally demonstrated to be NADPH-dependent. The core subunits of methylene-THF reductase (MTHFR) MetV and MetF are encoded downstream of Hdr- and Mvh-like genes (Supplementary Fig. 1). A similar genetic arrangement was shown before in *M. thermoacetica* and the MTHFR was proposed to be a hexaheteromeric complex of HdrCBA, MvhD and MetVF which uses NADH to reduce methylene-THF and a second so far unknown electron acceptor energetically similar to Fd^37^. For simplicity reasons, we assume Fd as second electron acceptor in our model. As mentioned earlier, CO_2_ reduction to CO requires Fd_red_. During growth on H_2_ + CO_2_, electrons are transferred from four H_2_ to two Fd and two NAD^+^ by the electron-bifurcating hydrogenase. One Fd_red_ is used for CO_2_ reduction by the Fdh. 0.5 NADH and Fd_red_ are used by the Stn complex for the reduction of one NADP^+^, which is required in the methyl branch for the reduction of methenyl-THF. 0.5 Fd_red_ are used by the Rnf complex for the reduction of 0.5 NADH, thereby generating a H^+^-gradient across the membrane. The resulting 0.5 NADH plus the left over 1.5 NADH from H_2_ oxidation are used by the MTHFR for the simultaneous reduction of methylene-THF and Fd. The reduced ferredoxin can then be used in the carbonyl branch for CO_2_ reduction. Next, CO and the methyl group are condensed to build acetyl-CoA. Further conversion finally yields one acetate from four H_2_ and two CO_2_. Since the net yield of ATP by substrate level phosphorylation in the WLP is zero, net ATP gain originates from the respiratory chain consisting of the Rnf complex and ATP synthase. Assuming a H^+^/ATP ratio of 4, 0.25 ATP can be synthesised by the H^+^-gradient (Fig. 5).

## Discussion

**Figure 6:**
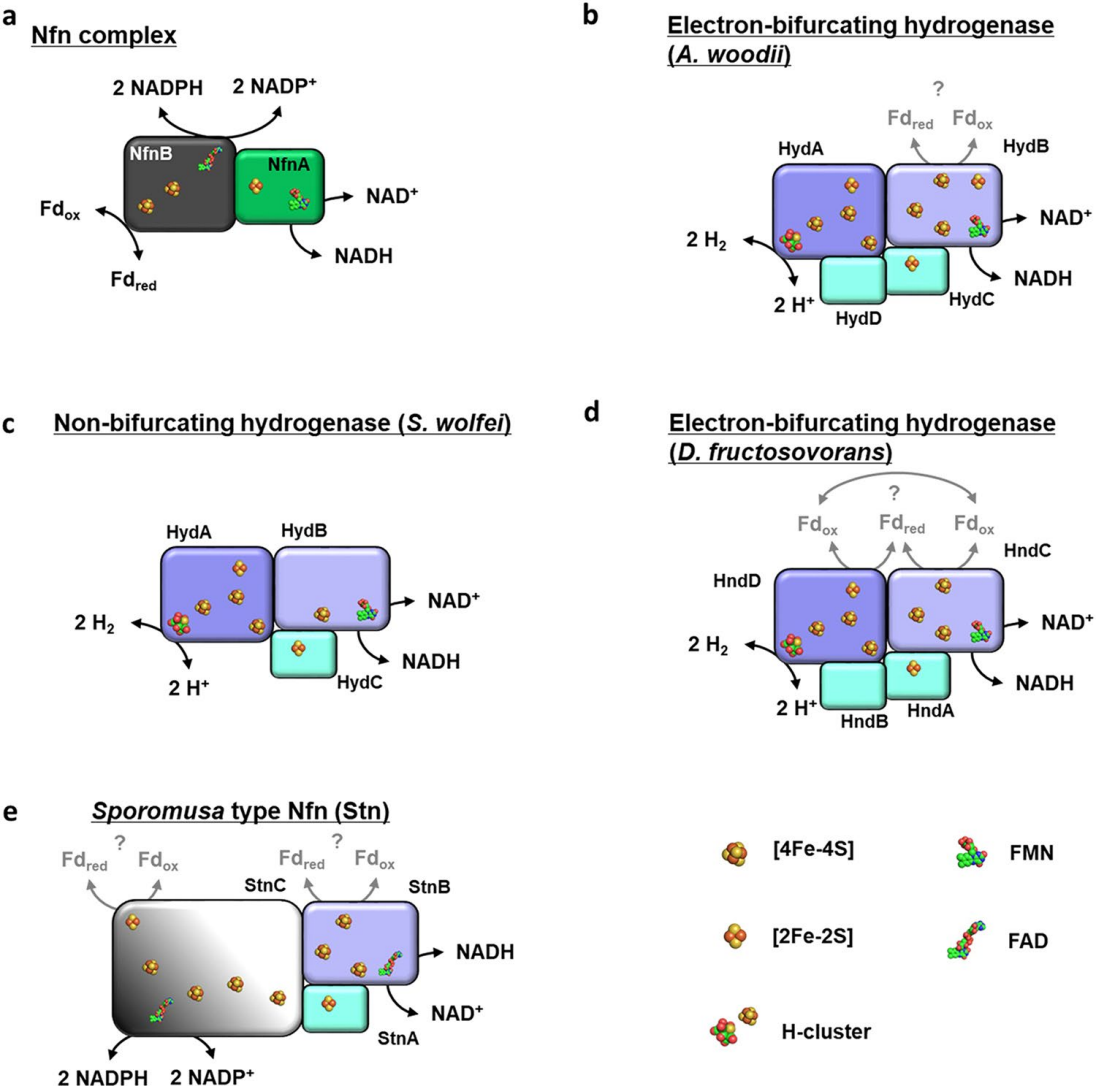
Model of Stn and homologous electron-bifurcating complexes. For explanations, see text. (**a**) The electron-bifurcating transhydrogenase Nfn is depicted. (**b**) The electron-bifurcating hydrogenase of *A. woodii* is depicted. (**c**) The non-bifurcating hydrogenase of *S. wolfei* is depicted. (**d**) The electron-bifurcating hydrogenase of *D. fructosovorans* is depicted. Fd is assumed to interact with HndC and HndD (**e**) The *Sporomusa* type Nfn is depicted. The FeS-strings were constructed arbitrarily.

Electron bifurcation is a mechanism to couple endergonic to exergonic redox reactions widely distributed in anaerobic bacteria. It is not involved in energy conservation but since ATP is not used as driving force for the endergonic reaction, it saves cellular ATP^38^. To date, eleven classes of electron-bifurcating enzymes that use the mechanism of FBEB are known^39^. In general FBEB is based on the feature of flavins to have three redox states^40^. During FBEB the bifurcating flavin is fully reduced by a 2e^−^ donor. In the fully reduced state the redox potential of the flavin/semiquinone couple is sufficient for the transfer of one electron to a high potential electron acceptor, leaving a flavin radical with an extremely negative redox potential. The highly reactive flavin radical is now able to reduce a second electron acceptor with a more negative redox potential than the first acceptor^38,41,42^. The Nfn complex is the most widely distributed electron-bifurcating enzyme and a well-studied specimen^43^. It has two subunits, NfnA and NfnB. NfnB harbours two [4Fe–4S]-clusters, the bifurcating FAD and the NADP(H) and ferredoxin binding sites. NfnA binds a [2Fe–2S]-cluster and an FMN and has the NAD(H) binding site. In the bifurcation mode, two electrons coming from NADPH are transferred to the bifurcating FAD. One electron travels via the [2Fe–2S]-cluster to the FMN in NfnA, leaving a low potential FADH°^-^ radical in NfnB, which is required for the reduction of the low potential electron acceptor ferredoxin via the two [4Fe–4S]-clusters of NfnB^31,44^. After a second cycle, the high potential electron acceptor NAD^+^ is reduced in a two electron transfer step from NfnA bound FMNH^-^ (Fig. 6a).

In contrast to Nfn complexes, little is known about the mechanism of electron bifurcation in electron-bifurcating hydrogenases. The core enzyme consists of three subunits, HydA, HydB and HydC^14^. The small subunit HydC coordinates one [2Fe–2S]-cluster. HydA is proposed to harbour the H-cluster for H_2_ oxidation, three [4Fe–4S]- and one [2Fe–2S]-cluster. HydB is proposed to coordinate three [4Fe–4S]- and one [2Fe–2S]-cluster as well and to harbour the FMN and the NAD(H) binding sites (Fig. 6b). Furthermore, comparison of HydB homologs of the electron-bifurcating hydrogenase of *A. woodii*^18^ and the non-bifurcating hydrogenase of *S. wolfei*^23^, suggested that either the [2Fe–2S]-cluster or one of the C-terminal [4Fe–4S]-clusters of HydB might be the region of Fd binding (Figs. 4, 6b, c). Meanwhile it was found that the hydrogenase of *D. fructosovorans* (Hnd), which also lacks the N-terminal [2Fe–2S]-cluster in the HydB homolog (HndC), is an electron-bifurcating enzyme^21^, leaving the C-terminal [4Fe–4S]-clusters of HydB as candidate for Fd binding (Figs. 4, 6d). In electron-bifurcating hydrogenases electrons are transferred from H_2_ to the FeS-cluster of the H-cluster in HydA, but the further electron flow is uncertain. One possibility is that electron-bifurcating hydrogenases harbour a second, electron-bifurcating flavin. If so, electrons are transferred from the H-cluster to the bifurcating flavin. Here, electrons are split up and two strings of FeS-clusters guide one electron to FMN in HydB and one electron to Fd, respectively^14,45^. Again, a second cycle is required to reduce NAD^+^ with electrons from FMNH^-^. Another mechanism suggests that the H-cluster is the site of electron bifurcation. In this model electrons are split up directly to two different FeS-strings, the first one leading to FMN in HydB and the second one to the Fd binding site^46^.

The Stn complex seems to be a hybrid built by homologs of HydC, HydB and a protein which is in part similar to NfnB. StnA and StnB are 49.6 and 59.9% identical to HydC and HydB of the bifurcating hydrogenase of *A. woodii*. Like HydC, StnA is predicted to bind a [2Fe–2S]-cluster. StnB is proposed to bind three [4Fe–4S]-clusters and a Flavin molecule and, therefore, harbours the NAD(H) binding site. Due to sequence analysis FMN was expected to be bound by StnB but only FAD was detected in the Stn complex by TLC. This is why we suggest an FAD molecule bound in StnB in our model (Fig. 6e). The third subunit is in part (amino acids 1–500) similar to the NADP(H)-binding subunit NfnB of Nfn complexes (e.g. 40.2 and 39.2% identity to SudA/NfnB of *P. furiosus* and *C. kluyveri*). StnC is proposed to bind at least four [4Fe–4S]-clusters, one [2Fe–2S]-cluster and one FAD (Supplementary Fig. 9) and recombinant StnC indeed binds FAD and oxidises NADPH. Contrary to NfnB, the [4Fe–4S]-cluster responsible for Fd reduction is not conserved in StnC. However, the C-terminal NuoG-like part of StnC adds further FeS-clusters to the complex, which might form a site for ferredoxin reduction. This is consistent with the presence of a conserved arginine residue in StnC (Arg239, Arg201 in SudA of *P. furiosus*; Supplementary Fig. 9), which is thought to have a major role in adjusting the low redox potential of the FADH°^−^/FAD pair needed for ferredoxin reduction in Nfn complexes^43,44^. Hence, we propose that the electrons from NADPH are transferred to the FAD bound by StnC first. Subsequently, the electrons are split up and shuttled via two distinct FeS-strings to FAD in StnB and the Fd bound by either StnC or StnB (Fig. 6e). The composition of the distinct FeS-strings and which FeS-cluster enables electron transfer from the Stn complex to ferredoxin has to be figured out in further studies, which might also shed light on the mechanism of electron-bifurcating hydrogenases.

The Stn complex exhibits NADH and Fd_red_-dependent NADP^+^ reduction and the NADPH-dependent reduction of NAD^+^ and Fd_ox_ and, therefore, links the cellular NADP(H) pool with the NAD(H) and the Fd pool. We here describe, that the Stn complex catalyses the energetically downhill reaction of NAD^+^ or NADP^+^ reduction with Fd_red_ and the reduction of NAD^+^ with NADPH. Electron bifurcation implies the strict need of two electron acceptors, however, low rates of the thermodynamic favourable electron transfer are reported for several electron-bifurcating enzymes. For example the electron-bifurcating hydrogenase of *T. maritima* shows H_2_ evolution when only Fd_red_ is available as electron donor^47^ and the Nfn complex of *C. kluyveri* catalyses electron transfer from Fd_red_ to NADP^+^^19^. We furthermore observed reduction of FMN or FAD with NADPH and a stimulating effect of both flavins in the bifurcation assay, indicating, that (depending on availability) one or the other flavin can be bound by the Stn complex, as already suggested for the electron-bifurcating hydrogenases of *M. thermoacetica* and *D. fructosovorans*^16,21^.

Inspection of genome sequences available revealed that Stn is widely distributed. Potential Stn-encoding gene clusters were found in Firmicutes, Proteobacteria and Synergistetes (Table 1). Furthermore, the before mentioned Nso complex^22^ is highly similar to the Stn complex and it seems reasonable that Nso might actually function as an NADH-dependent Fd_red_:NADP^+^ oxidoreductase in methanogenic archaea. The genome of *S. ovata* apparently only codes for the Stn complex but not the Nfn complex. Genome sequences of acetogens were screened and the presence of Stn and Nfn was found to be almost mutually exclusive (Table 2). Stn and Nfn distribution apparently does not follow a phylogenetic or a physiological trait. In 20 of 48 genomes of acetogens screened the *stn* gene cluster was found, whereas 27 of the genomes encode for one or more *nfn* gene clusters (Table 2). *A. woodii* has one *stn* cluster. Although it does not use NADPH as reductant in the WLP, NADPH is likely used as reductant in anabolic redox reactions. *Thermoacetogenium phaeum* has two different Stn-encoding gene clusters, whereas *C. carboxidivorans* and *C. magnum* have genes coding for an Stn complex and for several copies of Nfn complexes. Three of the genomes encode different Nfn complexes. The presence of multiple copies of Nfn encoding genes is also observed in species other than the acetogens. The archaeon *Pyrococcus furiosus* has two copies of Nfn^43,48^ and NfnI and NfnII were not only demonstrated to be differentially expressed (expression of NfnI is upregulated during sugar fermentation, whereas expression of NfnII is upregulated during sulfur reduction) but the purified Nfn homologs had different catalytic properties. NfnI was shown to transfer electrons from NADPH to ferredoxin and NAD^+^ simultaneously, whereas NfnII did not. NfnII exhibited Fd_red_:NADP^+^ oxidoreductase activity without addition of NADH but electron transfer from NADPH to Fd was not catalysed, indicating that NfnII might need a second, so far unknown electron acceptor to facilitate this endergonic electron transfer^48^. These results suggest that different transhydrogenases are important for the organism to deal with different substrate availability and living mode (sugar fermentation and sulfur reduction) and that the integration of Nfn or Stn encoding gene clusters into the genome (e.g. by lateral gene transfer) might enhance the range of potential habitats and, hence, contributes in increasing the fitness of the organism. The search for Stn homologs did also show that there are organisms like *C. drakei* and *T. primitia* which neither have genes coding for an Stn nor an Nfn complex, which raises the question if there is a third, so far unknown protein complex with a similar function in these organisms.

**Table 1:** Distribution of potential Stn-encoding gene clusters in bacteria and archaea.

Organism	Locus tag of StnC	% identitiy to StnC of *S. ovata*	Length [aa]	Phylum
*Acetoanaerobium sticklandii *DSM 519	CLOST_2294	45	1,195	Firmicutes
*Acetomicrobium mobile *DSM 13181	Anamo_1498	45	1,071	Synergistetes
*Alkaliphilus metalliredigens *QYMF	Amet_3096	44	1,187	Firmicutes
*Alkaliphilus oremlandii *OhILAs	Clos_0277	46	1,192	Firmicutes
*Ammonifex degensii *KC4	Adeg_0156	33	898	Firmicutes
*Caldicellulosiruptor changbaiensis *CBS-Z	Calcha_02442	51	1,178	Firmicutes
*Caldicellulosiruptor naganoensis *NA10	N907DRAFT_1313	50	1,178	Firmicutes
*Caldicellulosiruptor saccharolyticus *DSM 8903	Csac_0621	50	1,178	Firmicutes
*Dehalobacter restrictus *DSM 9455	Dehre_2348	32	891	Firmicutes
*Desulfitobacterium metallireducens *DSM 15288	Desme_0852	33	892	Firmicutes
*Desulfosporosinus acidiphilus *DSM 22704	Desaci_0777	31	892	Firmicutes
*Desulfosporosinus meridiei *DSM 13257	Desmer_0713	32	893	Firmicutes
*Desulfosporosinus orientis *DSM 765	Desor_0661	32	893	Firmicutes
*Desulfosporosinus *sp. BG	Ga0165177_101659	51	1,204	Firmicutes
*Desulfosporosinus *sp. OT	DOT_1508	51	1,204	Firmicutes
*Geoalkalibacter subterraneus *Red1	EJ58DRAFT_03287	45	1,192	Proteobacteria
*Methylomusa anaerophila *MMFC1	Ga0398860_479	76	1,184	Firmicutes
*Natranaerobius thermophilus *JW/NM-WN-LF	Nther_1249	37	1,073	Firmicutes
*Syntrophobotulus glycolicus *DSM 8271	Sgly_2685	31	894	Firmicutes
*Syntrophomonas palmitatica *JCM 14374	Ga0128340_1167	46	1,071	Firmicutes
*Terrisporobacter glycolicus *DSM 1288	G483DRAFT_1163	46	1,165	Firmicutes
*Thermococcus litoralis *DSM 5473	OCC_03032	41	955	Euryarchaeota
*Thermosediminibacter oceani *DSM 16646	Toce_0243	49	1,206	Firmicutes
*Thermovirga lienii *DSM 17291	Tlie_1249	37	1,241	Synergistetes

**Table 2:** Distribution of Stn and Nfn in acetogens.

Organism	Stn	Locus tag of StnC	% identity to StnC of *S. ovata*	Nfn	Locus tag of NfnAB	% identity to nfnB of *C. kluyveri*	% identity to nfnA of *C. kluyveri*	
*Acetitomaculum ruminis *DSM 5522	No	–	–	No	–	–	–	
*Acetoanaerobium noterae *ATCC 35199	Yes	EI48DRAFT_2626	45	No	–	–	–	
*Acetobacterium bakii *DSM 8239	Yes	Ga0100773_13321	42	No	–	–	–	
*Acetobacterium woodii *DSM 1030	Yes	Awo_c21410	42	No	–	–	–	
*Acetohalobium arabaticum *Z-7288, DSM 5501	No	–	–	Yes	Acear_0063/62	56	47	
*Acetonema longum *APO-1, DSM 6540	No	–	–	Yes	Alo_19762 + Alo_19767	62	48	
*Alkalibaculum bacchi *DSM 22112	Yes	Ga0244545_10455	47	No	–			
*Blautia coccoides *DSM 935	No	–	–	Yes	Ga0310518_10851/52	75	66	
*Blautia hydrogenotrophica *DSM 10507	Yes	RUMHYD_00360	40	No				
*Blautia producta *DSM 2950	No	–	–	Yes	G480DRAFT_02544/45	75	66	
*Blautia schinkii *DSM 10518	Yes	T506DRAFT_02634	45	No	–	–	–	
*Butyribacterium methylotrophicum *Marburg	Yes	BUME_01500	43	No	–	–	–	
*Calderihabitans maritimus *KKC1	Yes	Ga0302716_104267	47	No	–	–	–	
*Carboxydothermus ferrireducens *DSM 11255	No	–	–	Yes	CarfeDRAFT_00011620/10	67	55	
*Carboxydothermus hydrogenoformans *Z-2901	No	–	–	Yes	CHY_1991/92	67	56	
*Carboxydothermus pertinax *Ug1	No	–	–	Yes	Ga0346998_921/22	66	55	
*Clostridioides difficile *630 delta erm	No	–	–	Yes	Ga0098188_111728/27	76	66	
*Clostridium aceticum *DSM 1496	Yes	CACET_c07250	47	No	–	–	–	
*Clostridium autoethanogenum *DSM 10061	No	–	–	Yes	Ga0198697_113579	87	78	
*Clostridium carboxidivorans *P7	Yes	CcarbDRAFT_2642	51	4	–	–	–	
*Clostridium coskatii *PTA-10522	No	–	–	Yes	CLCOS_09810	87	78	
*Clostridium drakei *SL1	No	–	–	No	–	–	–	
*Clostridium formicaceticum *DSM 92	Yes	Ga0198698_11876	47	No	–	–	–	
*Clostridium kluyveri*	No	–	–	Yes	CKL_0459/60	100	100	
*Clostridium ljungdahlii *DSM 13528	No	–	–	Yes	CLJU_c37240	87	78	
*Clostridium magnum *DSM 2767	Yes	EJ33DRAFT_06085	52	2	EJ33DRAFT_02837/36, EJ33DRAFT_01727/26	82, 81	68,70	
*Clostridium methoxybenzovorans *DSM 12182	No	–	–	Yes	H204DRAFT_0416/17	76	66	
*Clostridium ragsdalei *P11	No	–	–	Yes	CLRAG_36680	87	78	
*Clostridium scatologenes *ATCC 25775	No	–	–	4	–	–	–	
*Eubacterium aggregans *SR12	No	–	–	Yes	Ga0073299_11488/87	76	65	
*Eubacterium callanderi *KIST612	Yes	ELI_3306	43	No	–	–	–	
*Eubacterium limosum *ATCC 8486	Yes	Ga0213646_111405	43	No	–	–	–	
*Holophaga foetida *TMBS4, DSM 6591	Yes	HolfoDRAFT_2931	36	No	–	–	–	
*Marvinbryantia formatexigens *I-52	No	–	–	Yes	Ga0056060_00161/60	76	65	
*Moorella glycerini *NMP	No	–	–	Yes	Ga0132330_10358/57	63	48	
*Moorella mulderi *DSM 14980	No	–	–	Yes	MOMU_22100/110	63	48	
*Moorella thermoacetica *ATCC 39073	No	–	–	Yes	Moth_1517/1518	62	49	
*Moorella thermoautotrophica *JW701/3	No	–	–	Yes	MTJW_15680/90	62	49	
*Oxobacter pfennigii *DSM 3222	Yes	Ga0068245_11828	57	No	–	–	–	
*Sporomusa acidovorans *DSM 3132	No	–	–	Yes	Ga0336822_563/62	71	68	
*Sporomusa malonica *DSM 5090	No	–		Yes	Ga0070592_12915/16	74	68	
*Sporomusa ovata *H1, DSM 2662	Yes	SOV_1c07760	100	No	–	–	–	
*Sporomusa silvacetica *DSM 10669	Yes	Ga0336821_2609	95	No	–	–	–	
*Sporomusa sphaeroides *E	No			Yes	SPSPH_02120/10	76	68	
*Terrisporobacter glycolicus *DSM 1288	Yes	G483DRAFT_1163	46	No	–	–	–	
*Thermacetogenium phaeum *DSM 12270	2	Tph_c21680 & c08060	45, 47	No	–	–	–	
*Thermoanaerobacter kivui *DSM 2030	No	–	–	Yes	Ga0069402_112324/23	87	53	
*Treponema primitia *ZAS-2	No	–	–	No	–	–	–	

## Methods

### Biochemicals and enzymes

NAD(P)^+^_,_ NAD(P)H, FAD, FMN, methyl viologen, benzyl viologen, glucose-6-phosphate, glucose-6-phosphatate dehydrogenase, tetrahydrofolate and formaldehyde were obtained from Sigma-Aldrich Chemie GmbH (Taufkirchen, Germany). Materials used for protein purification via FPLC were obtained from GE Healthcare, Sweden. Ferredoxin (a 2 × [4Fe–4S]-cluster ferredoxin) was purified from *C. pasteurianum*^49^, CODH was purified from *A. woodii*^11^.

### Cultivation of *S. ovata*

*S. ovata* DSM 2662 was grown under strictly anoxic conditions at 30 °C on DSM medium 311 with 20 mM fructose as carbon and energy source. Glycine betaine and Na_2_S × 9 H_2_O were omitted and 0.6 g/l Cystein-HCl × H_2_O was used as reducing agent, instead. Growth was monitored by measuring the optical density at 600 nm (OD_600_).

### Preparation of cytoplasm and membrane fraction

All buffers contained 2 mM DTE as reducing agent and 4 µM resazurin as redox indicator. Cells of *S. ovata* were harvested at late exponential growth phase (OD_600_ = 2) and washed twice with buffer A (50 mM Tris/HCl, pH 7.5; 20% Glycerin, 20 mM MgSO_4_, 5 µM FAD, 5 µM FMN). Cells were resuspended in buffer A, 0.5 mM PMSF and 0.1 mg/ml DNase I was added and the suspension was passed through a French pressure cell at 110 MPa. Cell debris was removed by centrifugation at 24,000×*g* for 45 min. Cytoplasmic- and membrane fraction were separated by centrifugation at 210,000×*g* for 45 min. For further use membrane fraction was washed twice in buffer A.

### Purification of *Sporomusa* type Nfn (Stn)

The cytoplasmic fraction was applied to a Q-Sepharose high performance column and eluted with a linear gradient from 0 to 400 mM NaCl. The Stn eluted between 150 mM NaCl and fractions with NADPH:MV oxidoreductase activity were pooled. (NH_4_)_2_SO_4_ was added to a final concentration of 2 M. The precipitate was removed by centrifugation (24,000×*g*, 30 min) and the supernatant was applied to a Phenyl-Sepharose HP column, equilibrated with buffer C (= buffer A + 2 M (NH_4_)_2_SO_4_). Elution was performed with a linear gradient from 2–0 M (NH_4_)_2_SO_4_ with buffer D (= buffer A + 250 mM NaCl). The activity eluted around 500 mM (NH_4_)_2_SO_4_. Fractions with activity were pooled, concentrated using ultrafiltration in 100-kDa Vivaspin tubes (Sartorius Stedim Biotech GmbH, Germany) and divided to avoid overloading of the following gel filtration on a Superdex 200 increase column (10/300 GL; GE-Healthcare). The gel filtration was equilibrated with buffer D and the Stn eluted at a volume of around 8.5 ml. Following, the sample was applied to a Blue-Sepharose HP column, which was equilibrated with buffer D. The Stn complex eluted by washing the column with 100% buffer B. The purified protein was stored in buffer A at 4 °C.

### Enzymatic activity assays

Except were indicated all enzyme activities were measured in anoxic glass cuvettes (d = 0.2 cm or 0.5 cm; Glasgerätebau Ochs, Germany) sealed by rubber stoppers in 100 mM Tris/HCl buffer (pH 7.5) under a N_2_-atmosphere. One unit is defined as transfer of 2 µmol electrons × min^−1^. All assays were performed in triplicates.

#### NAD(P)^+^ reduction with reduced ferredoxin (Rnf complex)

To determine the Rnf activity in the membrane fraction 30 µM ferredoxin were prereduced with CODH using a CO-atmosphere. 1 mM NAD(P)^+^ was added and the absorbance was followed at 340 nm (ε = 6.3 mM^-1^ × cm^−1^) to determine the reduction of NAD(P)^+^. The assay was performed in MOPS buffer (50 mM, pH 7) containing 10 mM NaCl and 20 mM MgSO_4_. Measurements were performed at 30 °C.

#### NAD(P)^+^ reduction with methylene-THF (methylene-THF dehydrogenase)

The methylene-THF dehydrogenase activity of the cytoplasmic fraction was determined by following the reduction of 0.5 mM NAD(P)^+^ with methylene-THF as electron donor. A racemic mixture of methylene-THF was produced non-enzymatically by adding 1.5 mM formaldehyde and 0.5 mM tetrahydrofolate to the buffer. Measurements were performed at 30 °C.

### Reactions catalysed by the Stn complex

#### Methyl viologen or benzyl viologen with NAD(P)H

Reduction of 5 mM MV with NADPH (0.5 mM) or NADH (0.5 mM) as electron donor was measured by following the absorbance at 604 nm (ε = 13.9 mM^−1^ × cm^−1^). If BV (5 mM) was used as electron acceptor the reduction of BV was measured by following the absorbance at 555 nm (ε = 12 mM^−1^ × cm^−1^). Measurements were performed at 20 °C.

#### FAD, FMN or ferricyanide reduction with NADPH

To determine the reduction of FAD (0.2 mM) or FMN (0.2 mM) with NADPH (0.25 mM) as electron donor, the absorbance of FAD (ε = 11.3 mM^−1^ × cm^−1^) or FMN was followed at 450 nm (ε = 12.5 mM^−1^ × cm^−1^). Reduction of ferricyanide (5 mM) with NADPH as electron donor was followed by measuring the absorbance at 420 nm (ε = 1 mM^−1^ × cm^−1^). Measurements were performed at 20 °C.

#### Simultaneous reduction of ferredoxin and NAD^+^ with NADPH

Reduction of ferredoxin and NAD^+^ with NADPH catalysed by the Stn complex was assayed at 50 °C in 50 mM Gly–Gly buffer (pH 8) containing 10 mM NaCl and 20 mM MgSO_4_. To keep the level of NADPH constant, 0.25 mM NADP^+^ were prereduced with 1 unit glucose-6-phosphate dehydrogenase and 20 mM glucose-6-phosphate (NADP^+^ reducing system) as reported earlier^19^. 30 µM ferredoxin and 0.5 mM NAD^+^ were used as electron acceptors. To determine the reduction of ferredoxin and the reduction of NAD^+^ the absorbance was followed at 430 nm (ε_Δox-red_ = 13.1 mM^−1^ × cm^−1^) and at 340 nm (ε = 6.3 mM^−1^ × cm^−1^), respectively. To determine the reduction NAD^+^ with NADPH, ferredoxin was omitted.

#### NADP^+^ reduction with NADH and reduced ferredoxin

NADP^+^ reduction catalysed by the Stn complex was assayed at 50 °C in 50 mM Gly-Gly buffer (pH 8) containing 10 mM NaCl and 20 mM MgSO_4_. 30 µM ferredoxin were prereduced with CODH using a CO-atmosphere. 0.25 mM NADH and 0.5 mM NADP^+^ was added and the change of absorbance at 340 nm was followed to determine NAD^+^ reduction. To determine NAD(P)^+^ reduction with reduced ferredoxin, NADH was omitted.

### Analytical methods

The protein concentration was determined according to Bradford^50^. Proteins were separated in 12% polyacrylamide gels and stained with coomassie brilliant blue G250. The iron content was determined calorimetrically according to Fish^34^. The nature of the flavin was determined as described before^51^.

### Heterologous expression StnA, StnB and StnC

The single subunits of the Stn complex were amplified using Phusion DNA polymerase (New England BioLabs; Ipswitch, MA) and genomic DNA of *S. ovata* as template. Primers used were: 5′-tttcatatgtggagccacccgcagttcgaaaaatctgcgtgtaattcgtgcgaaaaagagct-3′ and 5′-tttggatccctaaccttcacttacaacaccgcctttc-3′ for *stnA*, 5′-tttcatatgtggagccacccgcagttcgaaaaatctgcggtgaaggttagagtaggtcttgg-3′ and 5′-tttggatccctactcgatacaaactgcgtctaat-3′ for *stnB* and 5′-tttcatatgtggagccacccgcagttcgaaaaatctgcgagcaaaattagtataaatataaatggccgc-3′ and 5′-tttggatccctacaaactctgcctaccatttacaaaattc-3′ for *stnC*. The PCR products were cloned into the NdeI and BamHI restriction sites of pET21a and subsequently, the constructs were used to transform *E. coli* HB101. Plasmids were verified by DNA sequencing and introduced to *E. coli* BL21 (DE3) Δ*iscR*, which was already used for the successful production of iron sulfur-cluster containing proteins. Production and purification of the Stn subunits was performed as described earlier^35^.

## Supplementary information

Supplementary information.
