# Supplementary material for: The Sporomusa type Nfn is a novel type of electron-bifurcating transhydrogenase that links the redox pools in acetogenic bacteria
Source: Sci Rep. 2020 Sep 10;10:14872. doi: 10.1038/s41598-020-71038-2 (PMC7483475; doi:10.1038/s41598-020-71038-2)
Supplement: Supplementary file 1 — Supplementary information. [file 41598_2020_71038_MOESM1_ESM.pdf]

The *Sporomusa* type Nfn is a novel type of electron-bifurcating transhydrogenase that links the redox pools in acetogenic bacteria

SUPPLEMENTARY INFORMATION

Florian Kremp, Jennifer Roth and Volker Müller#

Department of Molecular Microbiology & Bioenergetics, Institute of Molecular Biosciences, Johann Wolfgang Goethe University, Max-von-Laue Str. 9, D-60438 Frankfurt, Germany

#Corresponding author.

Mailing address: Department of Molecular Microbiology & Bioenergetics, Institute of Molecular Biosciences, Johann Wolfgang Goethe University, Max-von-Laue-Str. 9, D-60438 Frankfurt, Germany.

Phone: 49-69-79829507. Fax: 49-69-79829306.

E-mail: vmueller@bio.uni-frankfurt.de.

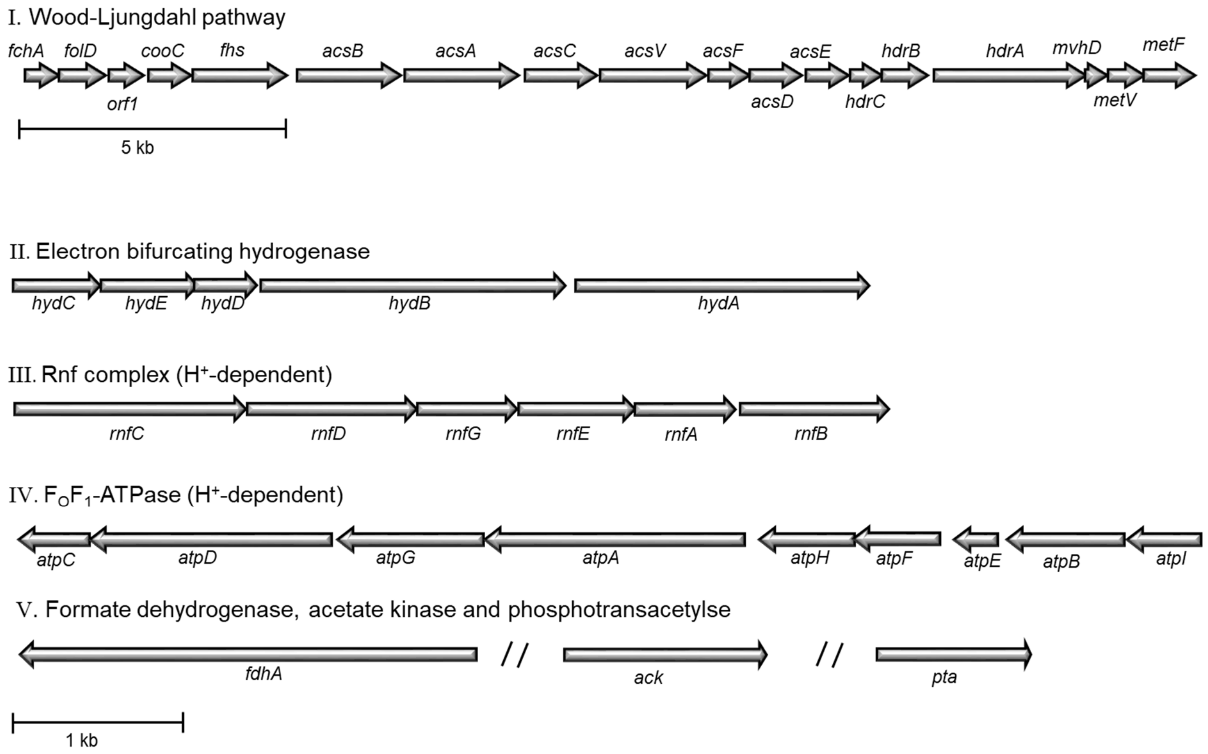

Supplementary Figure 1. Gene clusters encoding the central metabolism of *S. ovata*. Gene cluster I contains the genes for methenyl-THF cyclohydrolase (*fchA*), the methylene-THF dehydrogenase (*folD*), the formyl-THF synthetase (*fhs*) and the methylene-THF reductase (*metVF*). Further the CODH/ACS (*acsA* and *acsB*), the corrinoid/iron sulfur protein (*acsCD*), a corrinoid activation and regeneration protein (*acsV*), the CoFeSP methyltransferase (*acsE*), the CODH maturation factor (*Cooc*) and the CODH nickel-insertion protein (*acsF*) are encoded in gene cluster I, as well as the genes *hdrCBA* and *mvhD* which share similarities to subunits of the heterodisulfide reductase and methyl viologen reducing hydrogenase from methanogenic archaea. A hypothetical protein is encoded by *orf1*. Gene cluster II contains the electron-bifurcating hydrogenase (*hydCEDBA*). Gene cluster III contains the genes encoding a H^+-dependent Rnf complex (*rnfCDGEAB*) and gene cluster IV contains the genes *atpIBEFHGDC* which code for a H^+-dependent F_OF_1-ATP synthase. In V the genes coding for a formate dehydrogenase (*fdh*), the acetae kinase (*ack*) and the phosphotransacetylase (*pta*) are depicted.

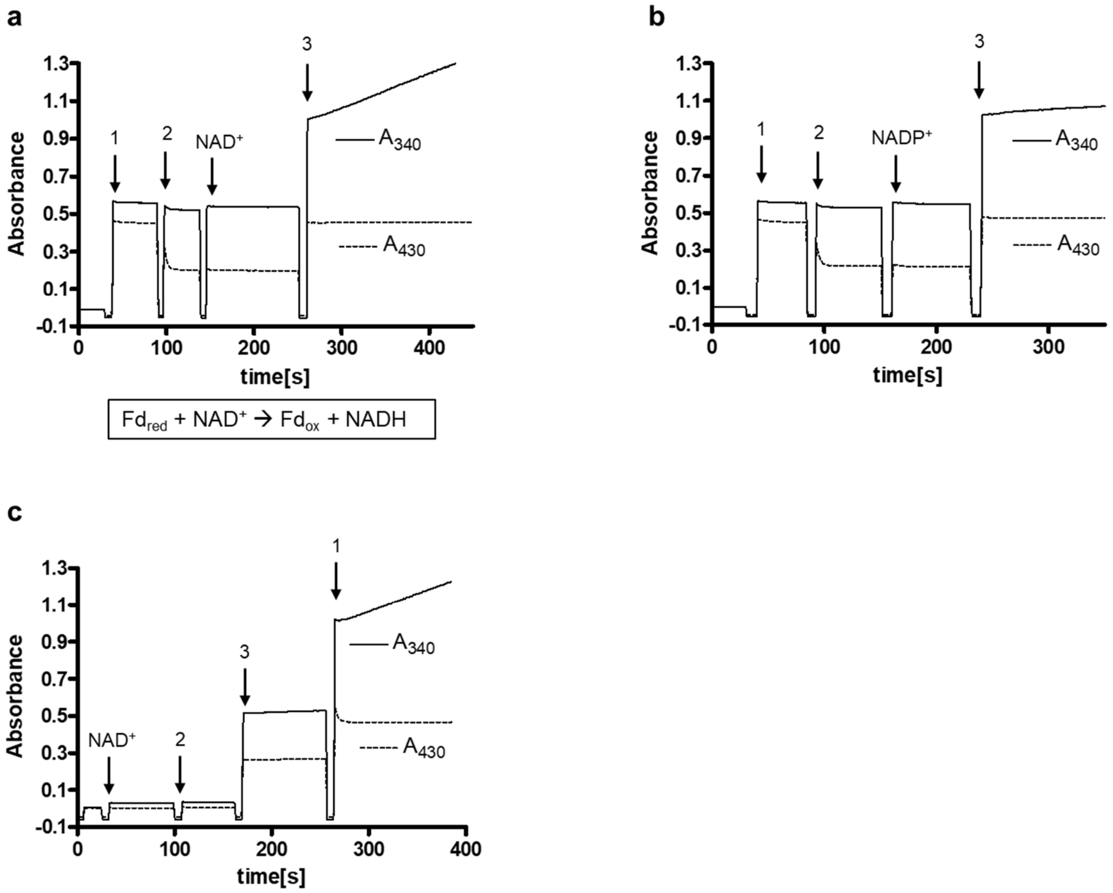

Supplementary Figure 2. Measurement of the Rnf activity in the membrane fraction of *S. ovata*. The Rnf activity of the membrane fraction was measured in 50 mM MOPS, pH 7, containing 10 mM NaCl and 20 mM $MgSO_4$. (a) 30 µM Fd were added to the buffer (1) and reduced with CO by purified CODH from *A. woodii* (2). Reduction of Fd was followed by measuring the absorbance at 430 nm. After addition of 1 mM NAD^+. The reaction was started by adding the membrane fraction to the assay (3). The reduction of NAD^+ was measured by following the absorbance at 340 nm. (b) The enzyme assay was performed as in (a) but $NADP^+$ rather than NAD^+ was used as electron acceptor. (c) The assay was performed as in (a) but ferredoxin was used to start the assay.

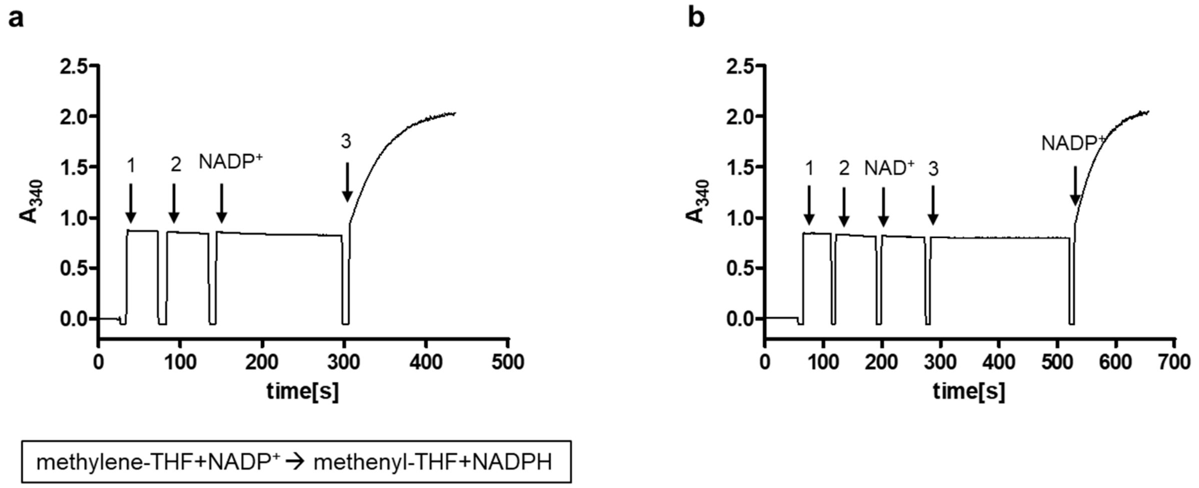

Supplementary Figure 3. Measurement of the methylene-THF dehydrogenase activity in the cytoplasmic fraction of *S. ovata*. The MTHFDH activity of the cytoplasmic fraction was measured in 100 mM Tris/HCl, pH 7.5. (a) 0.5 mM THF and 1.5 mM formaldehyde were added to the buffer to form methylene-THF non-enzymatically (1, 2). After the addition of 1 mM $NADP^+$ the measurement was started by adding cytoplasmic fraction to the assay (3). The reduction of $NADP^+$ was measured by following the absorbance at 340 nm. (b) The assay was performed as in (a) but NAD^+ was used as an electron acceptor. As a control $NADP^+$ was added after no increase of A_{340} (NAD^+ reduction) was observed.

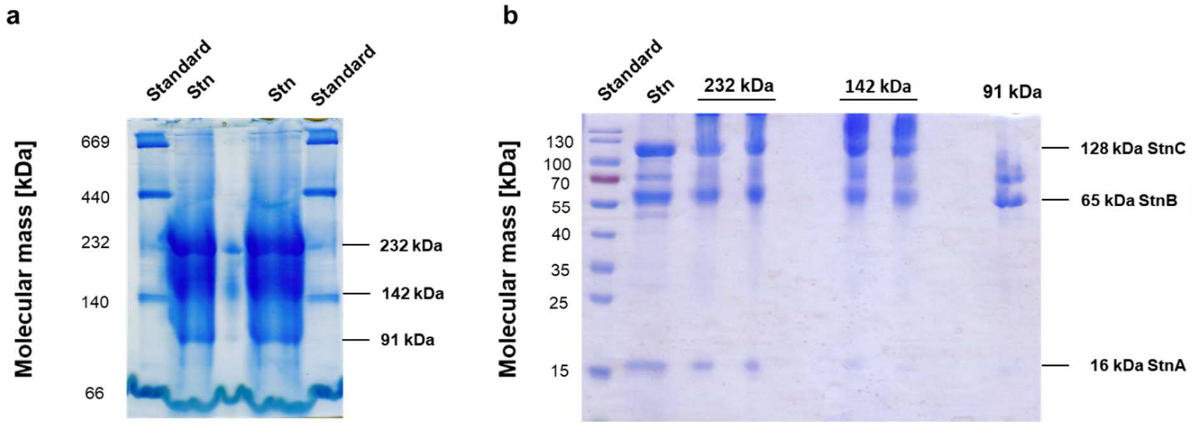

Supplementary Figure 4. Native- and subsequent SDS-PAGE of the purified Stn complex. The preparation of the Stn complex was separated by native PAGE (a) and the protein complexes with molecular masses of 232, 142 and 91 kDa were cut out of the gel and further separated by denaturating SDS-PAGE (b) together with 10 µg of the purified Stn complex (Stn).

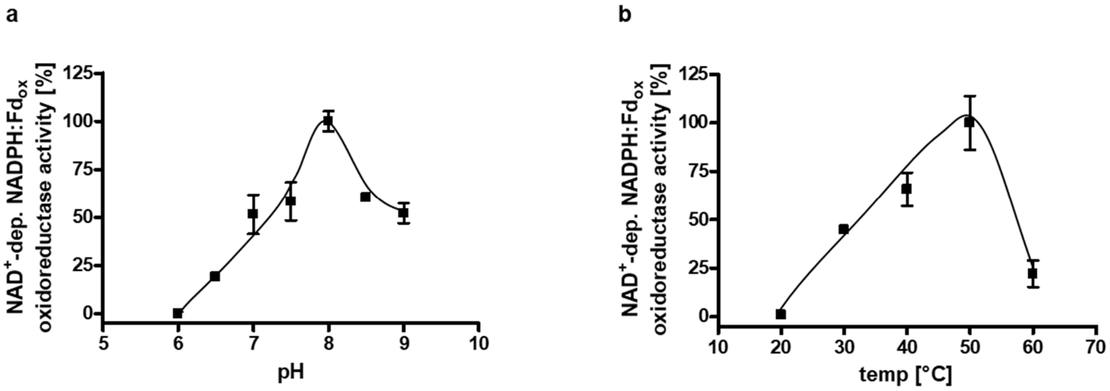

Supplementary Figure 5. pH and temperature optima of the Stn complex. Simultaneous reduction of NAD^+ and Fd_{ox} with NADPH was measured as described in methods. To determine the pH optimum of the Stn complex a combined buffer containing 25 mM MES/MOPS/Tris/CHES, 10 mM NaCl and 20 mM $MgSO_4$ was used with different pH values as indicated and the measurements were performed at 30°C (a). To determine the temperature optimum of the Stn complex the measurement was performed in 50 mM Gly-Gly buffer, pH 8 (b). 100% corresponds to 1.6 (a) and 3.5 (b) µmol ferredoxin reduced x min^{-1} x mg^{-1}. All data points are mean ± SD (n = 3).

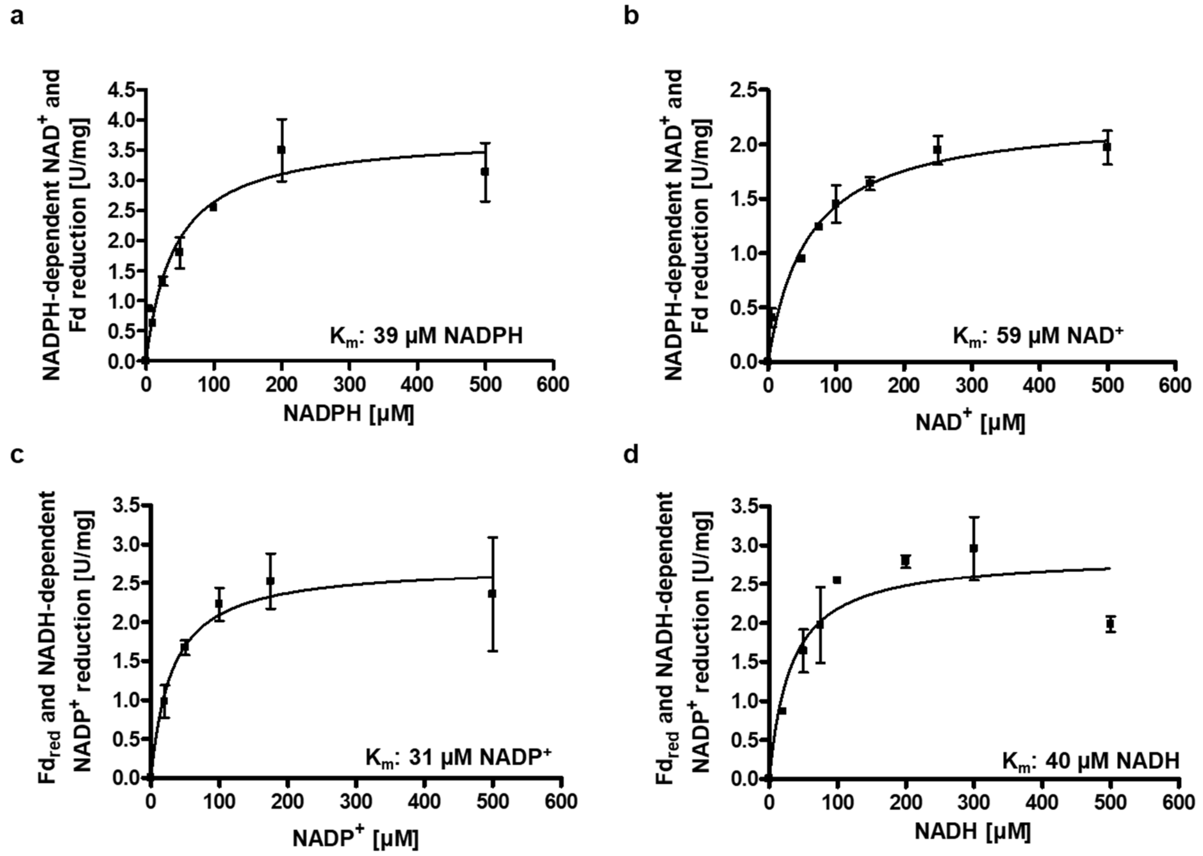

Supplementary Figure 6. K_m-value determination of the simultaneous reduction of NAD^+ and Fd with NADPH and for the Fd_{red}- and NADH-dependent reduction of $NADP^+$. The assays were performed as described in methods using varying amounts of either NADPH (a), NAD^+ (b), $NADP^+$ (c) or NADH (d). All data points are mean ± SD (n = 3).

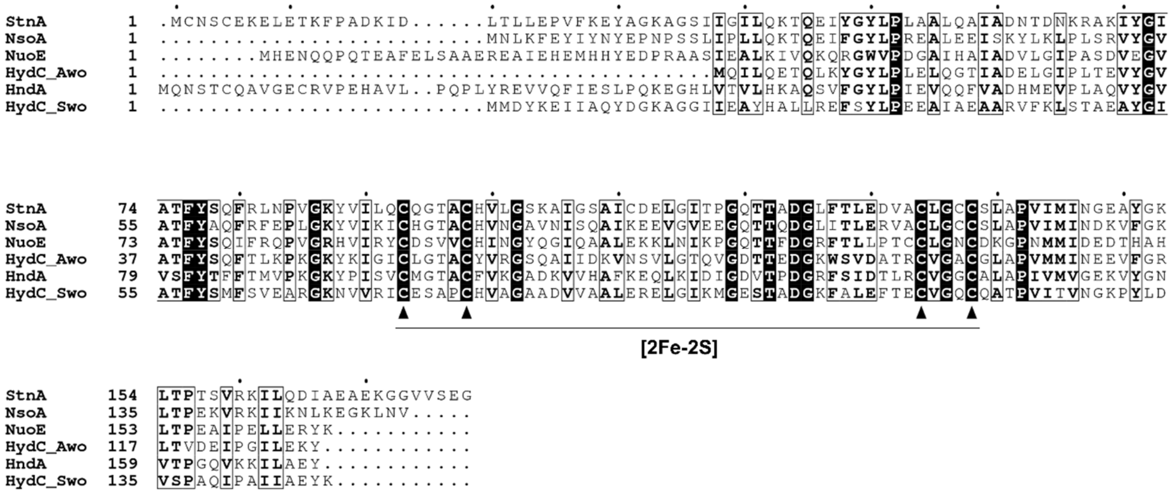

Supplementary Figure 7. Sequence alignment of StnA and homologs. Black triangles indicate amino acids responsible for coordinating the FeS-cluster. White characters with black background show strictly conserved amino acids. Bold black characters indicate amino acids with similar characteristics. Sequence alignments were performed with ClustalOmega multiple sequence alignment and visualized with ESPript 3.0[1].

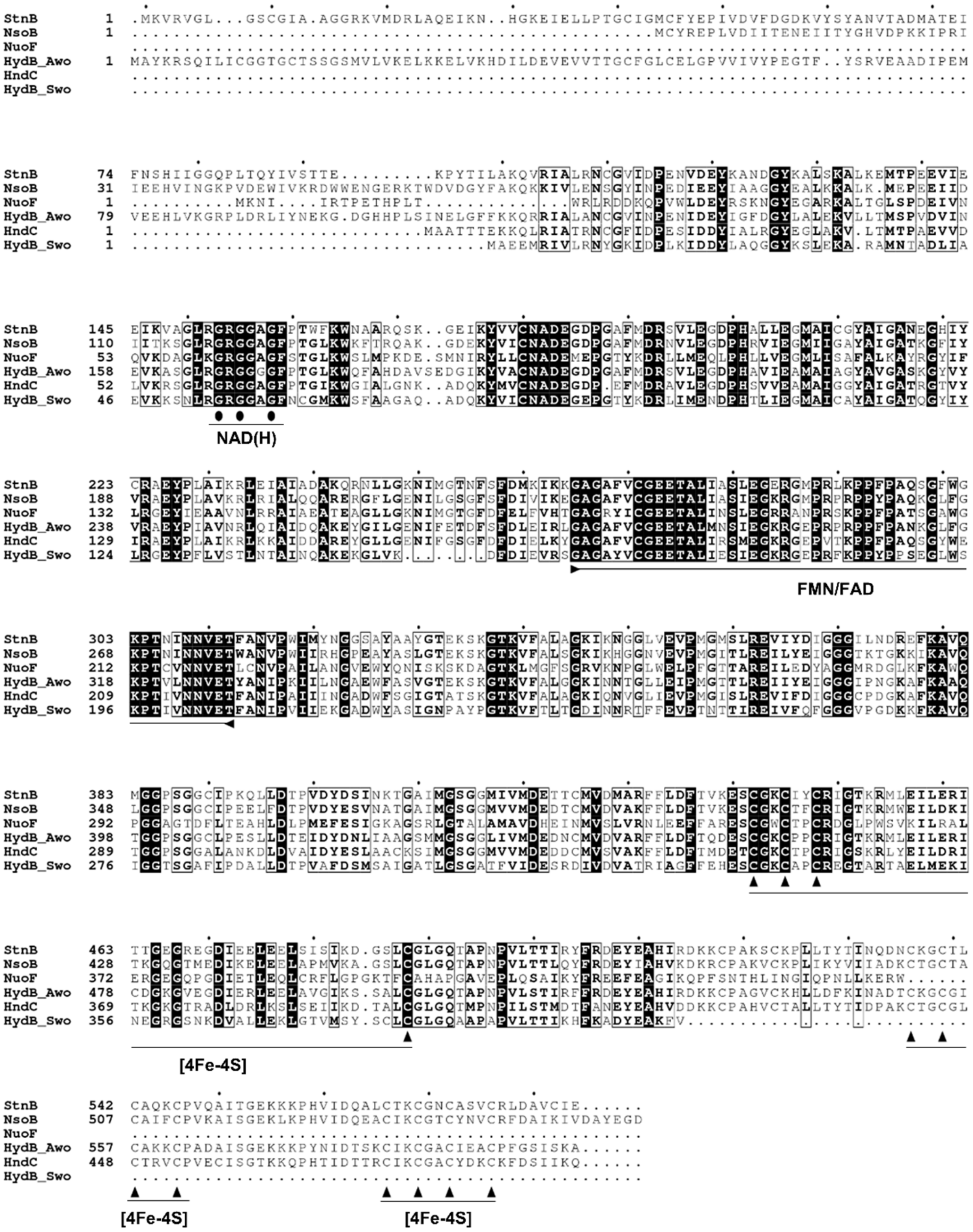

Supplementary Figure 8. Sequence alignment of StnB and homologs. Black circles indicate the conserved glycines of the NADH-binding motif. The FMN binding site is indicated. Black triangles indicate amino acids responsible for coordinating the FeS-clusters. White characters with black background show strictly conserved amino acids. Bold black characters indicate amino acids with similar characteristics. Sequence alignments were performed with ClustalOmega multiple sequence alignment and visualized with ESPript 3.0[1].

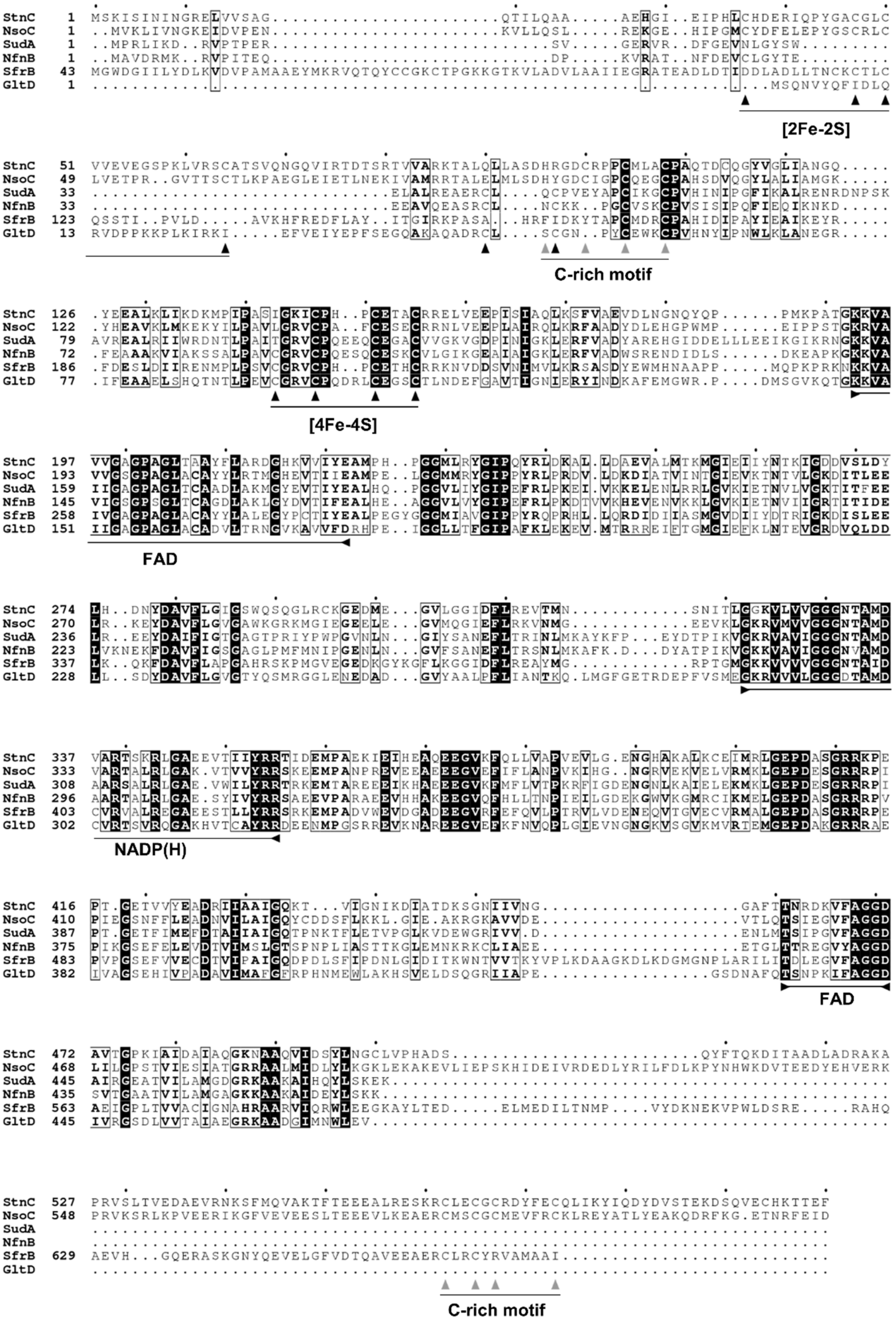

Supplementary Figure 9. Sequence alignment of the N-terminal sequence of StnC and homologs. The FAD- and NADP(H)-binding sites are indicated. Grey triangles indicate residues of cysteine-rich motifs known from NsoC. Black triangles indicate amino acids responsible for coordinating the FeS-clusters. White characters with black background show strictly conserved amino acids. Bold black characters indicate amino acids with similar characteristics. Sequence alignments were performed with ClustalOmega multiple sequence alignment and visualized with ESPript 3.0[1].

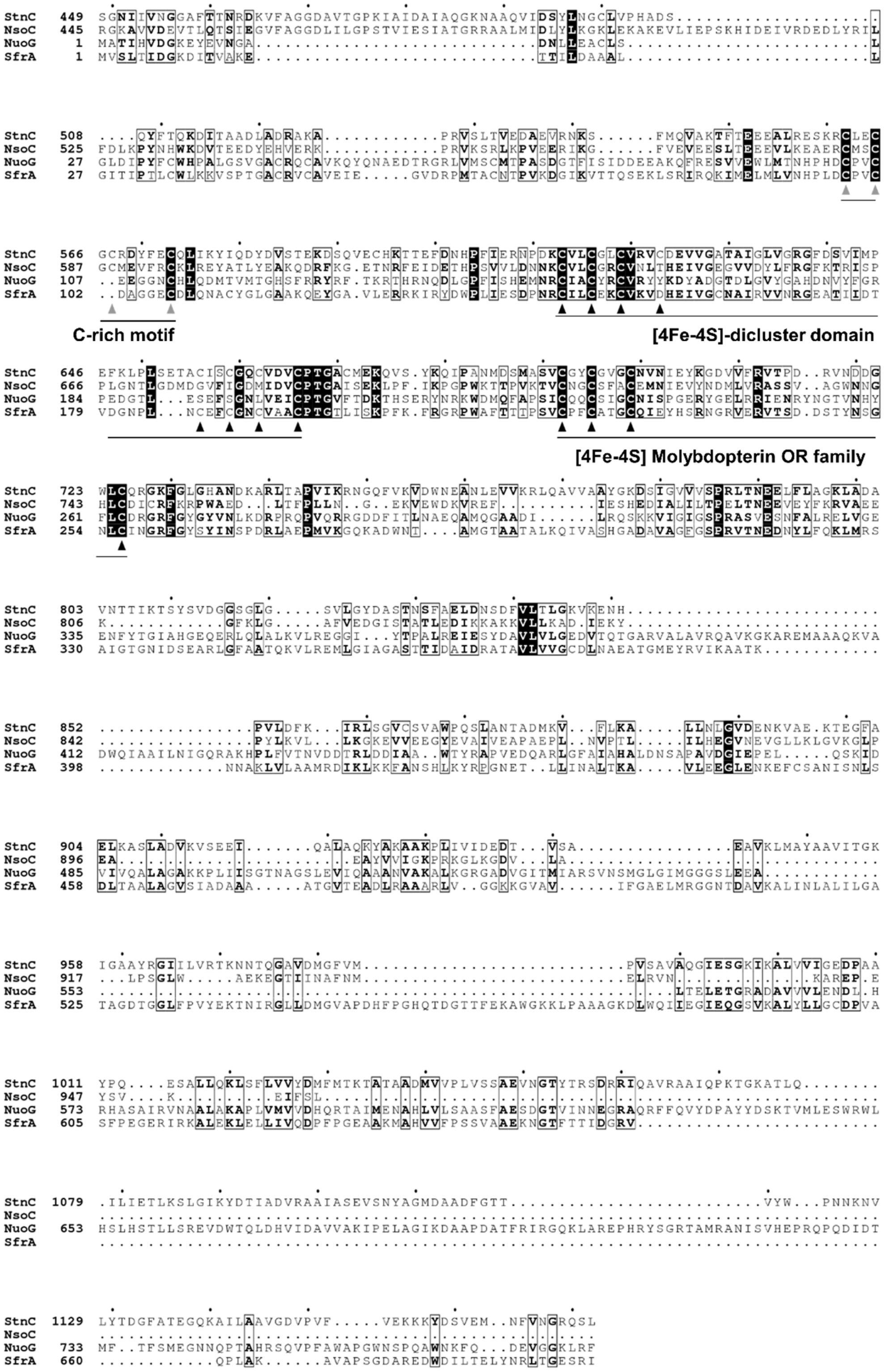

Supplementary Figure 10. Sequence alignment of the C-terminal sequence of StnC and homologs. Grey triangles indicate residues of cysteine-rich motifs known from NsoC. Black triangles indicate amino acids responsible for coordinating the FeS-clusters. White characters with black background show strictly conserved amino acids. Bold black characters indicate amino acids with similar characteristics. Sequence alignments were performed with ClustalOmega multiple sequence alignment and visualized with ESPript 3.0[1].

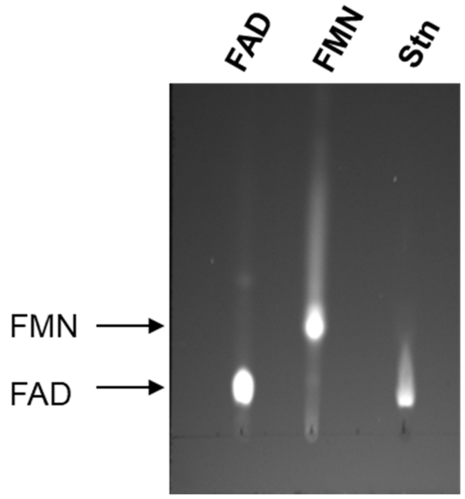

Supplementary Figure 11. Thin layer chromatography of flavins extracted from the Stn complex. The flavin-content of ~1 nmol purified Stn complex was loaded onto the TLC plate, 1 nmol FAD and FMN were used as standards. Separation of the flavins was achieved by using 60% [v/v] *n*-butanol, 15% [v/v] glacial acetic acid and 25% [v/v] H_2O as the mobile phase.

Supplementary Table 1. Purification of the Stn complex of *S. ovata*.

	Protein [mg]	NADPH: MV_{ox} OR [U*/mg]	Total activity [U]	Yield [%]	Purification [x-fold]
**Cytoplasm**	1127	3.0	3381	100	1.0
**Q-Sepharose**	361	11.3	4079	120	3.7
**Phenyl-Sepharose**	65	35.7	2321	69	11.9
**Superdex 200**	8	217.5	1740	51	72.5
**Blue-Sepharose**	6	278.2	1669	49	92.7

*1 Unit is defined as 1 µmol NADPH oxidised per minute.

Supplementary References

1 Robert, X. & Gouet, P. Deciphering key features in protein structures with the new ENDscript server. *Nucleic Acids Res.* **42**, W320-324 (2014).
